# The effect of sesamol on endogenous substances and oxidative stability of walnut oil

**DOI:** 10.3389/fnut.2024.1476734

**Published:** 2024-10-17

**Authors:** Qin Cheng, Yuanyuan Bao, Qi Lin, Tingmei Qi, Xinyong Zhang

**Affiliations:** ^1^College of Food Science and Technology, Yunnan Agricultural University, Kunming, Yunnan, China; ^2^College of Agronomy and Biotechnology, Yunnan Agricultural University, Kunming, Yunnan, China

**Keywords:** walnut oil, sesame oil, sesamol, endogenous antioxidants, fatty acids

## Abstract

This study explored the effect of sesamol on the stability of walnut oil based on the changes of endogenous characteristics in the oxidation process, which provided a theoretical reference for the application of natural antioxidants in walnut oil. A total of 300 mg/kg sesamol (SP), compound antioxidant AC (sesamol 353.62 mg/kg, citric acid 149.60 mg/kg, and BHA 76.33 mg/kg) and 35% sesame oil (35%-SO) were added to walnut oil respectively; in addition, 200 mg/kg t-butylhydroquinone (TBHQ), butylated hydroxyanisole (BHA), and citric acid were used as controls and blank walnut oil to study their effects on peroxide value, acid value, carbonyl compounds, conjugated olefins, phenols, flavonoids, sterols, vitamin E, *β*-carotene, and 51 fatty acids of walnut oil and their correlation between endogenous antioxidant components. The results showed that the addition of SP, 35%-SO, and AC could inhibit the increase of peroxide value, acid value, and carbonyl compounds in walnut oil, and could inhibit the decrease of *β*-carotene, total phenols, total sterols, and vitamin E. SP and 35%-SO could inhibit the decrease of total flavonoids, and several antioxidants could inhibit the decrease of endogenous antioxidant components in walnut oil. At the same time, it can better inhibit the change of unsaturated fatty acids in walnut oil. By the end of oxidation, the unsaturated fatty acids of blank walnut oil decreased by 10.31%, but AC, SP, and 35%-SO treatment groups increased by 10.90, 5.09 and 4.13%, respectively. Indicating that it had a certain protective effect on unsaturated fatty acids in walnut oil. There was a certain correlation between the endogenous substances of walnut oil. so the addition of several antioxidants can enhance the endogenous antioxidants of walnut oil, inhibit the oxidation of unsaturated fatty acids, and inhibit the increase of carbonyl compounds, codienes, acid value, and peroxide value. SP and AC have better antioxidant effects on walnut oil and improve the stability of walnut oil.

## 1 Introduction

Walnut oil is a vegetable oil with high unsaturated fatty acid content (1). It has a variety of physiologically active functions, such as anti-inflammatory (2), hypolipidemic (3), and antioxidant (4) properties. Walnut oil is not only rich in fatty acids but also contains a variety of endogenous antioxidants, so it has highly valuable for both consumption and nutrition (5). The main unsaturated fatty acids in walnut oil are linoleic acid (60.42–65.77%), oleic acid (13.21–19.94%), and linolenic acid (7.61–13%) (6). The oils with higher unsaturated fatty acids are easily oxidized and rancid (7). The content of vitamin E in walnut oil was 302.46–462.10 mg/kg, the content of polyphenols was 44.47–64.01 mg/kg (8), the content of total sterols was 1,207–2,026 mg/kg (9), and the content of total flavonoids was 3.20–24.12 mg/g (10). Walnut oil is easy to be oxidized and rancid during storage, which will produce peroxide and free fatty acids, resulting in the increase of peroxide value, acid value and carbonyl compounds (11). The oxidation stability of oil is not only closely related to the fatty acid composition, but also related to the trace components in the oil. The trace concomitants contain both pro-oxidation and antioxidation substances. Tocopherol has the highest degree of inhibition of primary oxidation of walnut oil, whereas total phenols have the best effect on secondary oxidation inhibition (12). Various sterols can be oxidized and degraded by themselves, and carotenoids, polyphenols, flavonoids, and other nonlipids can delay the oxidation of oil in walnut oil, whereas conjugated olefins and carbonyl compounds have pro-oxidation effects (13).

Sesamol, also known as 3,4-methylenedioxyphenol, white or white-like to light gray crystalline powder is a natural antioxidant active ingredient, which is naturally present in sesame oil and has been shown to be one of the reasons why sesame oil has more antioxidant capacity than other vegetable oils (14, 15). Sesamol is a natural antioxidant active ingredient and the antioxidant effect is greater than that of other bioactive components sesamin and sesamin (16). It has antioxidant (17), anti-inflammatory (18), antiaging (19), antihyperglycemia (15), anticancer (20) and other biological activities. Therefore, sesamol, as a natural antioxidant, can improve the oxidative stability of walnut oil.

Due to the high content of unsaturated fatty acids in walnut oil, such as linseed oil, linoleic acid and oleic acid, so it is easy to be oxidized and rancid during processing, transportation and storage. Resulting in a decline in its quality, and even the production of small molecular aldehydes, ketones, carbonyls and other compounds endangering human health. It is very important to ensure the quality of walnut oil and extend its shelf life (21). Synthetic antioxidants such as propyl gallate (PG), dibutyl hydroxytoluene (BHT), TBHQ, BHA, etc. are widely used in edible oil storage, but their potential hazards have been questioned. Therefore, it is a new trend to find safe, low-toxic and effective natural antioxidants to prevent oil oxidation (22). If the peroxide value reached the limit value (peroxide value ≤0.25 g/100 g) in the national standard of vegetable oil as the evaluation index, the predicted shelf life of blank walnut oil was 160 days (23).

On the basis of the previous research results of the research group, sesamol, compound antioxidants and sesame oil of 35% were added to walnut oil, and blank walnut oil was used as blank control. Using TBHQ, BHA, and citric acid as the control, the effects of several antioxidants on the oxidative stability indexes such as peroxide value, acid value, carbonyl compounds, conjugated olefins, flavonoids, polyphenols, sterols, *β*-carotene, tocopherols, and fatty acids in oil culture medium were studied. The oxidative stability of the composite oil was evaluated by evaluating the synergistic effect of endogenous antioxidant components, which provided a theoretical reference for the application of natural antioxidants in walnut oil.

## 2 Materials and methods

### 2.1 Materials and equipment’s

#### 2.1.1 Materials

The walnuts were collected from the YangBi county of Dali (Yunnan, China). The walnut variety is “Pao walnut,” which was harvested during the 2021–2022 season. The walnuts after harvest were transported to the laboratory, dried in an oven at 40°C for 72 h and processed to walnut oil immediately. Walnut shells were broken by hand, and the kernels were separated from the shells before processing. Sesame was purchased from Laishui County Jingu Grain and Oil Food Co., Ltd. and production place Inner Mongolia, China. which was harvested during the 2021–2022 season.

Sesame and walnut were pressed by screw cold press, (Ranger oil press, RG-306, Shenzhen Xiangju Intelligent Co., Ltd.) then the two oils were centrifuged and filtered to obtain crude oil, respectively. The oil after centrifugation was collected to clarify no impurities. They were sealed at a low temperature and were removed room light for later use.

#### 2.1.2 Equipment’s

Digital display constant temperature water bath cooker (HH-8), Beijing Changfeng Instrumentation Co., Ltd.; high performance liquid chromatograph (1020), Agilent; ultraviolet spectrophotometer (UV-1800), Aoyi Instrument (Shanghai) Co., Ltd.; gas chromatograph Trace (1300) Thermo; multi tube vortex mixer (BE-2600), Qilinbeier Instrument Manufacturing Co., Ltd.; mass spectrometer (ISQ7000), Thermo.

### 2.2 Methods

#### 2.2.1 Oil and antioxidant preparation

According to the results of the previous experiment and National food safety standard-Standard for the use of food additives, the compound antioxidant (AC), 300 mg/kg sesamol (SP), 35% sesame oil (35%-SO) were added to walnut oil, and the blank walnut oil was used as the control. Compared with 200 mg/kg TBHQ, BHA, and citric acid, all samples were placed in a constant temperature drying oven at 60°C ± 1°C for accelerated oxidation for 10 days, and samples were taken every 2 days. SP extraction: sesame oil was heated at 160°C for 20 min to obtain the extraction raw material. The ratio of material to liquid was 1:3.9 g/mL, the extraction time was 2 h, and the extraction temperature was 54°C to obtain the crude extract. The extract SP was the crude extract. The remaining oil was removed with n-hexane, then anhydrous ethanol (1:8 g/mL) was added and stirred at 60°C until dissolved and crystallized. Oil oxidation temperature and time: Accelerated oxidation was carried out according to the Schaal oven method. According to the Schaal oven method, 1 d was equivalent to 16 days storage at 25°C. Oxidation time: According to the preliminary experimental results, the peroxide value of all experimental groups exceeded the national standard after oxidation for more than 10 days, and there was no need to continue the experiment.

#### 2.2.2 Determination of peroxide value of walnut oil

Refer to GB/5009.227–2016 determination. 2.50 g oil samples were put into a 250 mL iodometric bottle, added with 30 mL trichloromethane-glacial acetic acid solution (3: 2), shaken well to completely dissolve, added with 1.00 mL potassium iodide saturated solution, plugged tightly, shaken for 0.5 min, and placed in the dark for 3 min. Take out and add 100mLwater, shake well, and titrate with 0.01 mol/L sodium thiosulfate standard solution.

#### 2.2.3 Determination of acid value of walnut

Refer to GB/5009.229-2016 determination. The 20 g oil samples were shaken well with 50 mL ether-isopropanol solution (1: 1) and 3–4 drops of 1 g/100 mL phenolphthalein indicator in a 250 mL conical flask and titrated with 0.1 mol/L sodium hydroxide solution.

#### 2.2.4 Determination of carbonyl compounds of walnut oil

Refer to GB/5009.230–2016. Weigh 0.1 g sample in test tube with plug, add 5 mL benzene dissolved oil sample, 3 mL 4.3 g/100 mL trichloroacetic acid solution and 5 mL 50 mg/100 mL 2,4-dinitrophenylhydrazine solution to shake well, heat in a 60°C water bath for 30 min, take out and cool to room temperature, then add 10 mL 4 g/100 mL potassium hydroxide-ethanol solution, place for 10 min, and measure the absorbance at 440 nm.

#### 2.2.5 Determination of *β*-carotene

According to the method of Ellison (24), 1 mL of oil sample was diluted with petroleum ether to a 10 mL volumetric flask, and the blank was made without oil sample solution. The absorbance was measured at 450 nm with spectrophotometric techniques.

#### 2.2.6 Determination of tocopherol

According to the method of Cheruth (25), 1 mL of oil sample was taken in a 10 mL volumetric flask, and 5 mL of chloroform, 3.5 mL of 0.07% 2,2′-bipyridine, 0.5 mL of 0.2% ferric chloride Fecl3 were added. The volume was scaled, mixed evenly, stood for 1 min, and the absorbance was measured at 520 nm.

#### 2.2.7 Determination of phenols

According to the method of Mokhtar (26), a 2.5 g oil sample + 2.5 mL anhydrous ethanol solution was extracted, fully oscillated, and centrifuged, and the supernatant was collected to obtain the polyphenol extract. A 0.5 mL extract +2.5 mL Folin–Ciocalteu reagent (0.1 mol/L), 2.0 mL 7.5% sodium carbonate solution, fully mixed, reacted in the dark for 1 h, and the absorbance was measured at 765 nm.

#### 2.2.8 Determination of sterols

According to the method of Jiang (27). The sample was extracted with 0.5 mL sample + 5 mL anhydrous ethanol, and 2 mL supernatant was taken in the colorimetric tube, then 2 mL anhydrous ethanol and phosphorus–sulfur iron color reagent were added, respectively. The color was developed for 15 min, and the absorbance was measured at 520 nm.

#### 2.2.9 Determination of flavonoids

According to Muzolf (28) and other methods, 1 mL sample was taken in a 25 mL volumetric flask, then 1 mL 5% NaNO_2_, 1 mL 10% Al (NO_3_) 3, and 10 mL 4% NaOH were added, respectively. The sample was diluted with absolute ethanol to the scale, mixed, and stood for 15 min, and the absorbance was measured at 360 nm.

#### 2.2.10 Determination of conjugated olefins

According to the method of Wong (29), 0.25 g of oil sample was taken in a 10 mL volumetric flask, and the absorbance was determined at 233 nm and 268 nm, respectively, with cyclohexane as blank zeroing.

#### 2.2.11 Determination of fatty acid content in walnut oil

The changes of fatty acids in walnut oil samples at 0 h and the end of oxidation (peroxide value >10 mmol/L) were determined, and the relationship between fatty acids and oxidative stability was analyzed. All samples were placed in a constant temperature drying oven at 60°C ± 1°C for accelerated oxidation. The peroxide value was measured every 2 days, and the peroxide value was more than 10 mmol/L for sampling.

##### 2.2.11.1 Sample preparation

See Table 1 for details.

**Table 1:** Sample preparation.

Samples	Initial oxidation	End of oxidation
walnut oil	HO	HE
sesame oil	SO	SE
AC	HO-AC	HE-AC
35%-SO	HO-35%	HE-35%
SP	HO-SP	HE-SP
TBHQ	HO-TBHQ	HE-TBHQ
BHA	HO-BHA	HE-BHA
citric acid	HO-NMS	HE-NMS

The initial stage of oxidation was 0 days, and the oxidation end of walnut oil, sesame oil, AC, 35%-SO, SP, TBHQ, BHA and citric acid was 2 days, 20 days, 8 days, 8 days, 6 days, 12 days, 4 days and 4 days, respectively.

##### 2.2.11.2 Fatty acid standard curve making

The mixed standard solution of 51 fatty acid methyl esters (4,000 μg/mL) was prepared with n-hexane into 10 mixed standard concentration gradients of 1 μg/mL, 5 μg/mL, 10 μg/mL, 25 μg/mL, 50 μg/mL, 100 μg/mL, 250 μg/mL, 500 μg/mL, 1,000 μg/mL, 2000 μg/mL, where the concentration is the total concentration of each component. The mother liquor is stored at minus 20°C, and the working standard solution is ready for use.

##### 2.2.11.3 Fatty acid extraction

Refer to the method of HovingLR (30). Appropriate amount of samples were taken in a 15 mL centrifuge tube; accurately add 2 mL 1% sulfuric acid methanol solution, fully mix and shake for 1 min; esterification in 80°C water bath for 30 min; after cooling, accurately add 1 mL of n-hexane to the extract, shake and mix for 30 s, stand for 5 min, then add 5 mL of H_2_O (4°C) to wash, centrifuge at 4°C for 10 min at 3,500 rpm; 700 μL supernatant was accurately sucked into a 2 mL centrifuge tube, and then 100 mg anhydrous sodium sulfate powder was added to remove excess water. The mixture was oscillated and mixed for 30 s, and centrifuged at 12,000 rpm for 5 min. Of the supernatant, 50 μL was accurately extracted into a 2 mL centrifuge tube and diluted with 950 μL of n-hexane. Then 100 μL diluent was accurately drawn into a 2 mL centrifuge tube, and 900 μL n-hexane was added to dilute. After accurately aspirating 300 μL diluent into a 2 mL centrifuge tube, 15 μL 500 ppm methyl salicylate was added as the internal standard, the mixture was oscillated and mixed for 10 s, and 200 μL supernatant was added to the detection bottle.


$$\mathrm{Sample content}=C\times 1\times \left(200/\mathrm{sampling volume}\right)\times 100$$

Note: content unit, μg/mL; C unit, μg/mL; sampling unit, μL.

##### 2.2.11.4 Chromatographic and mass spectrometric conditions for the determination of fatty acids

Refer to the experimental method of Beccari (30) and Hoving (31). Chromatographic conditions include ThermoTrace1300 (Thermo Fisher Scientific, United States) gas phase system, and chromatographic column ThermoTG-FAME capillary column (50 m × 0.25 mmID × 0.20 μm); the injection volume was 1 μL, and the split ratio was 8:1. The inlet temperature was 250°C, the ion source temperature was 300°C, and the transmission line temperature was 280°C. The initial temperature of programmed heating was 80°C and maintained for 1 min. The temperature was raised to 160°C at 20°C/min for 1.5 min. Then the temperature was raised to 196°C at 3°C/min for 8.5 min. Finally, the temperature was raised to 250°C at 20°C/min for 3 min. The carrier gas was helium with a flow rate of 0.63 mL/min. Mass spectrometry conditions consist the ThermoISQ7000 mass spectrometer (Thermo Fisher Scientific, United States), electron impact ionization source, SIM scanning mode, and electron energy 70 eV.

### 2.3 Data statistics and analysis

The standard deviation ± mean is used to express all data. SPSS 25.0 and Microsoft Excel were used for statistical analysis of the data. The analysis employed Duncan’s multiple comparison method, which is based on one-way analysis of variance. The minimal threshold of significance was established at *p* < 0.05 and *p* < 0.01 in each instance. The Origin 2021 program was used to plot the outcome.

## 3 Results and analysis

### 3.1 Effect of sesamol on oxidation degree of walnut oil

**Figure 1:**
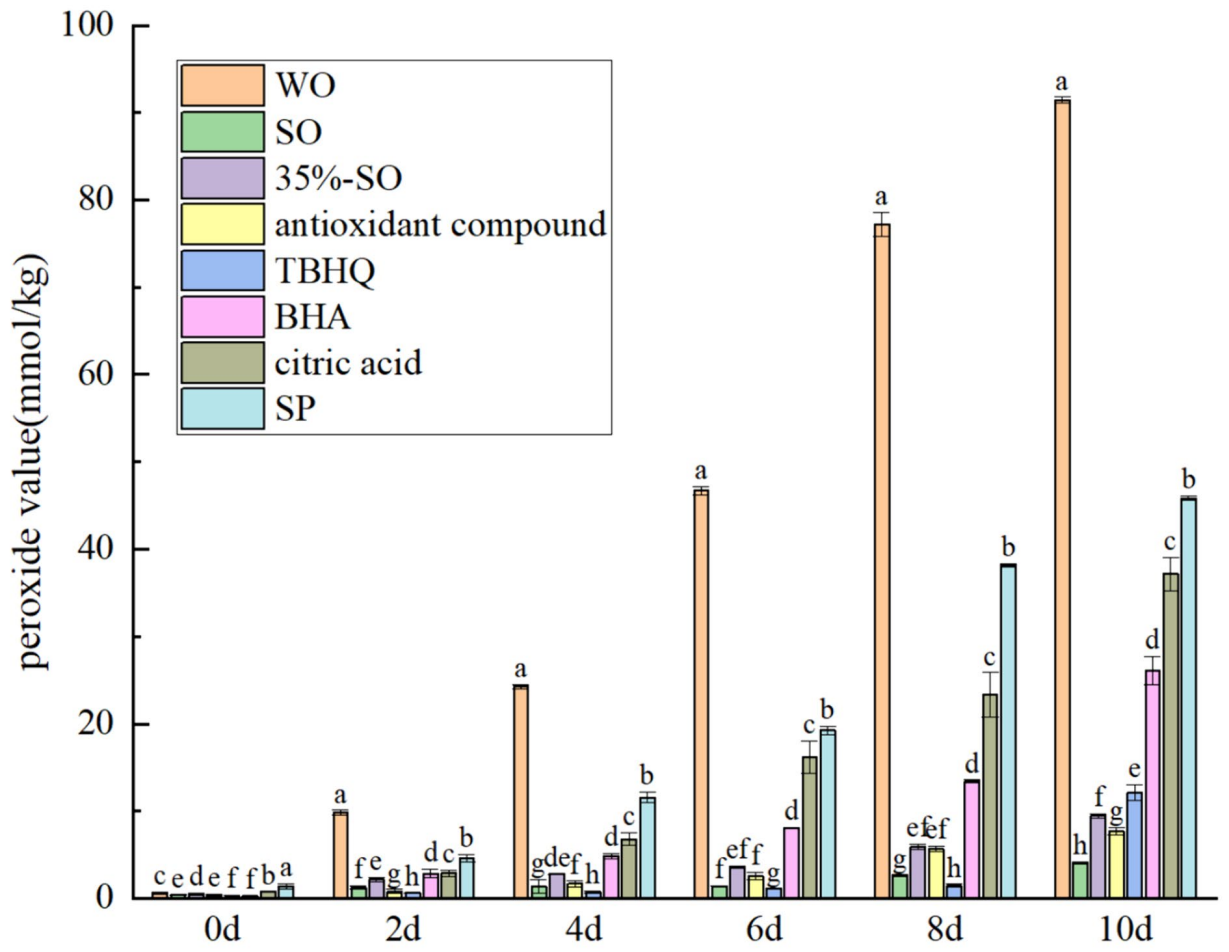
Effects of sesamol on peroxide value of walnut oil.

**Figure 2:**
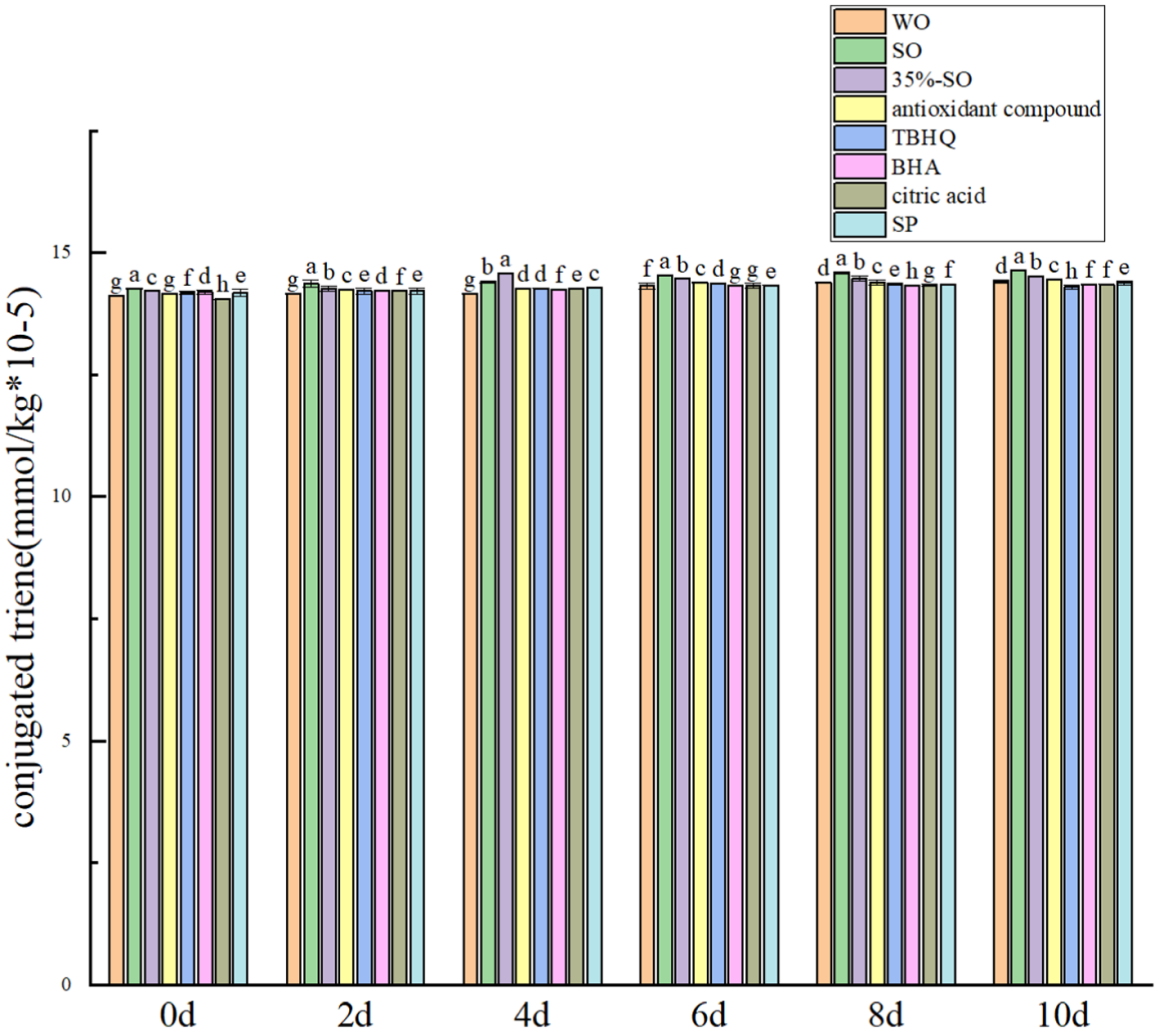
Effects of sesamol on conjugated triene in walnut oil.

During the oxidation process, the peroxide value, acid value, carbonyl compounds and conjugated olefin content of walnut oil changed with the oxidation time. So it can be used to reflect the effect of sesamol on the oxidation degree of walnut oil. Peroxide value is an index of oil oxidation process (32). The peroxide value, acid value, carbonyl compounds and conjugated olefin of all experimental groups increased with the extension of oxidation period (Figures 1, 2).

On the 10th day, the peroxide value of blank walnut oil was the highest, and the peroxide value of sesame oil was the lowest (Figure 1). It can be seen that sesame oil is not easy to be oxidized compared with walnut oil, and its stability is stronger than walnut oil. Sesame oil of 35% was mixed with walnut oil, and it was found that the peroxide value was significantly lower than that of blank walnut oil (*p* < 0.05). It shows that the addition of sesame oil can inhibit the increase of peroxide value of walnut oil, which may be because the oxidation stability of sesame oil is stronger than that of walnut oil (33). The oxidation stability of walnut oil is stronger than that of walnut oil when it is compounded with other oils. It may be caused by the antioxidant activity of endogenous antioxidant components in another oil and the mixing of unsaturated fatty acids, which reduces the degree of unsaturation (34). Sesame oil contains a variety of endogenous antioxidant components, and the content of unsaturated fatty acids in sesame oil is lower than that in walnut oil (35). The addition of extract SP could significantly inhibit the increase of peroxide value in walnut oil (*p* < 0.05). After the addition of AC the peroxide value was significantly lower than that of blank walnut oil group and SP group, indicating that the AC could better inhibit the oxidation of walnut oil and improve the oxidation stability of walnut oil. The peroxide value of all treatment groups was significantly lower than that of the blank group (p < 0.05), and the peroxide value was in the order of blank walnut oil > SP > citric acid > BHA > TBHQ >35%-SO > AC, indicating that it could inhibit the oxidative rancidity of walnut oil, thus prolonging its shelf life, and the effect of AC was stronger than that of other treatment groups. Peroxide value is an important evaluation index of primary oxidation products, which reflects the peroxide content of primary oxidation products in oil. During storage, the peroxide values of all sample groups showed a trend of increasing first and then decreasing, which was consistent with the rule that peroxides were generated in large quantities and then partially degraded in different oxidation stages of oil (36).

**Figure 3:**
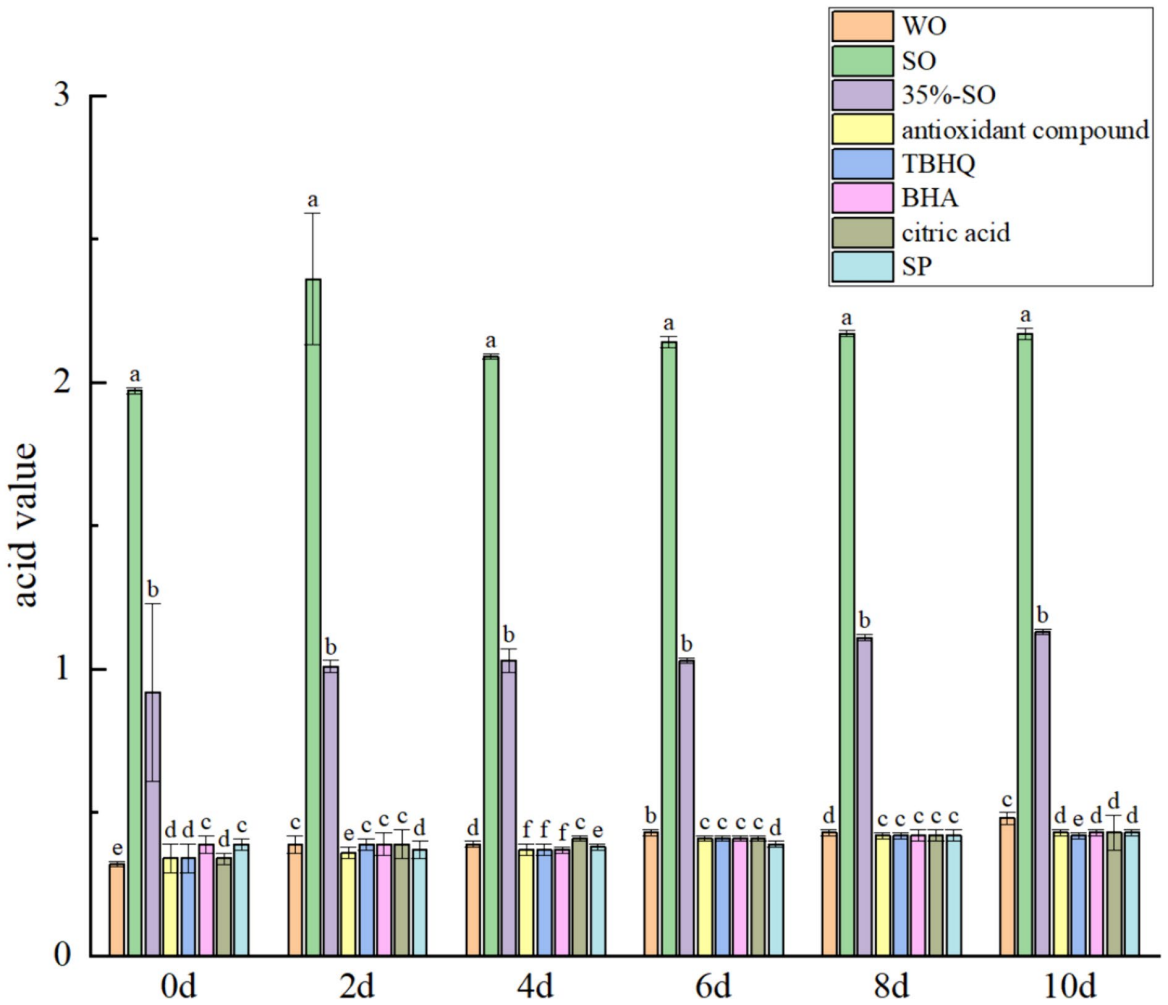
Effect of sesamol on acid value of walnut oil.

With the extension of oxidation time, the acid value of all treatment groups increased slowly. and the increase of the blank group was the largest (Figure 3). The acid value of sesame oil was the highest, followed by 35 % sesame oil, probably because the higher acid value of sesame oil is related to the oil crop itself (37). The acid value after adding several antioxidants was lower than that of the blank group, it shows that several antioxidants can prevent the production of free fatty acids in walnut oil, thereby inhibiting the increase of acid value, but the overall increase of acid value was small. Because the oxidation cycle was short, the primary oxidation products did not further degrade into small molecular carbonyl compounds, such as aldehydes, ketones, acids, and other substances (38).

**Figure 4:**
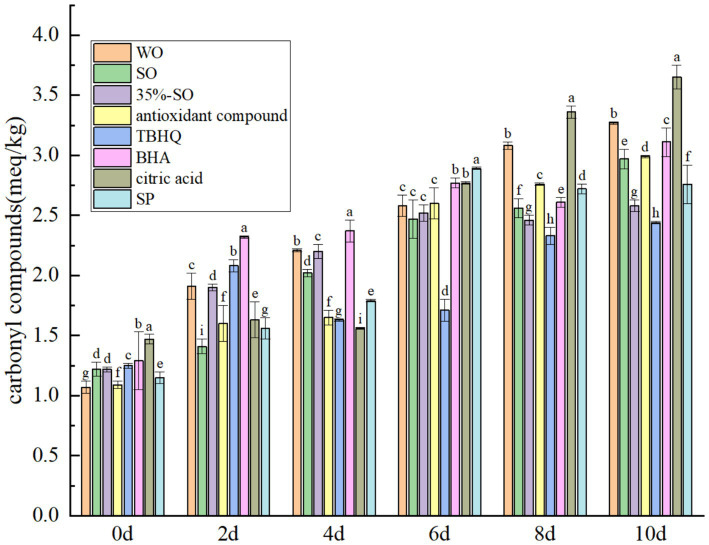
Effects of sesamol on carbonyl compounds in walnut oil.

Carbonyl compound is an important secondary oxidation product, which can be used as an important index of edible oil quality. With the prolongation of accelerated oxidation time, the carbonyl compounds in all treatment groups increased (Figure 4), Because during oil oxidation, carbonyl compounds are produced (39). On the 10th day, the carbonyl compounds in the blank group were significantly higher than those in the sesame oil group, which further indicated that walnut oil was easier to oxidize than sesame oil. The carbonyl compounds in the extract SP group were significantly lower than the blank walnut oil group (*p* < 0.05), which shows that it can inhibit the production of carbonyl compounds. The order of carbonyl compounds was as follows: citric acid > walnut oil > BHA > AC > sesame oil > SP > 35%-SO > TBHQ, and the other groups were lower than the blank walnut oil group (except citric acid), indicating that the addition of the above antioxidants can inhibit the production of carbonyl compounds, thus having an antioxidant effect on walnut oil.

**Figure 5:**
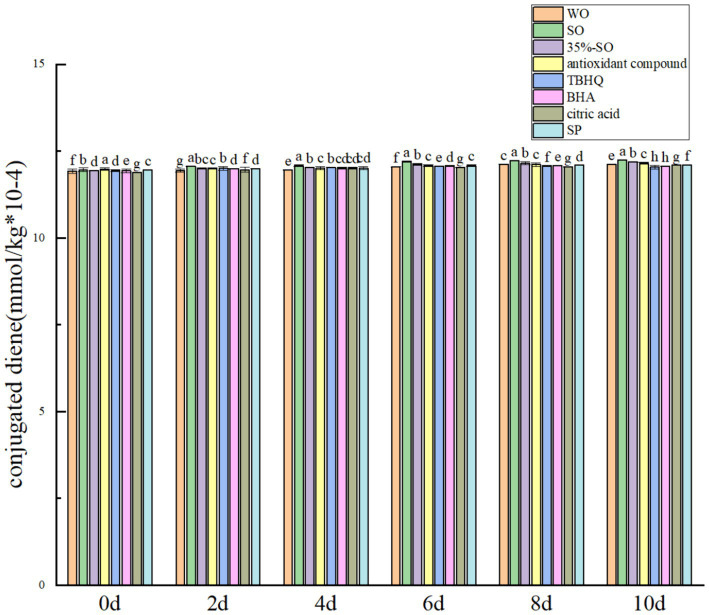
Effects of sesamol on conjugated diene in walnut oil.

Conjugated olefin is the primary product index in the process of oil oxidation. The degree of primary oxidation of oil can be expressed by conjugated olefin value and carbonyl value, respectively. That is, the higher the content of conjugated olefin, the deeper the degree of oil oxidation. With the extension of accelerated oxidation time, the contents of conjugated diene and conjugated triene are gradually increasing (Figures 2, 5). The reason may be that the peroxide generated during the oxidative rancidity of walnut oil is unstable. The oxidation of methylene double bond interrupted the diene bond of oil (40), but there was no significant difference in the increase range. It may be that the oxidation period is short, so its content changes little. In summary, the addition of sesamol and sesamol compound antioxidants can inhibit the increase of peroxide value, acid value carbonyl compounds and conjugated olefins, indicating that they can delay the oxidation of walnut oil and improve its stability.

### 3.2 Effect of sesamol on endogenous concomitant antioxidants in walnut oil

**Figure 6:**
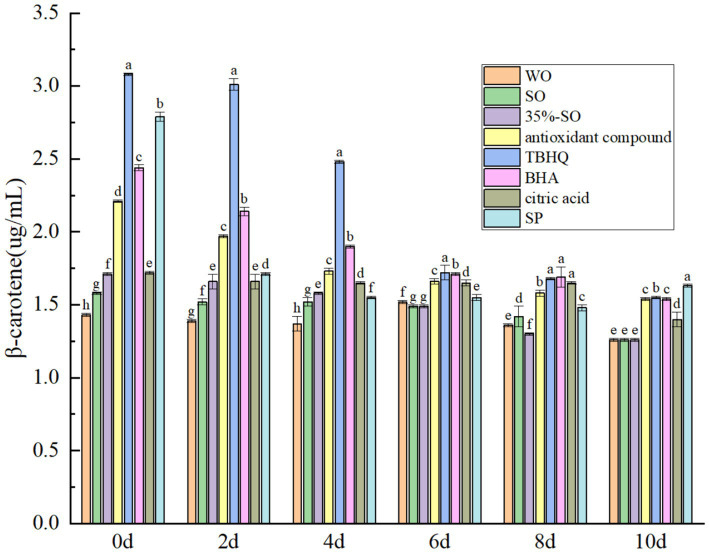
Effects of sesamol on β-carotene in walnut oil.

The endogenous antioxidant substances in walnut oil mainly include polyphenols, flavonoids, sterols, vitamin E, and daucosterol. These substances can exert antioxidant effects in oil and inhibit the oxidation of oil. *β*-carotene is a naturally occurring fat-soluble food functional component with an antioxidant effect (41). As the accelerated oxidation time prolongs, the content of *β*-carotene gradually decreased (Figure 6), which may be attributed to the fact that *β*-carotene has many conjugated double bonds and is oxidized during accelerated oxidation (42). On the 10th day, the content of *β*-carotene in sesame oil and walnut oil was lost, and there was no significant difference, while the content of *β*-carotene in SP was the highest, which was higher than that of TBHQ. The results showed that the effect of SP on *β*-carotene in walnut oil was stronger than that of TBHQ. AC also had a good protective effect on β-carotene, second only to TBHQ, indicating that the addition of SP and AC could better protect the decrease of β-carotene in walnut oil. It shows that the addition of SP can prevent the oxidation of β-carotene, which is beneficial to the stability of walnut oil. After adding the several antioxidants, the content of β-carotene was; SP > TBHQ > AC = BHA > citric acid >35%-SO = sesame oil = walnut oil. It shows that the addition of several antioxidants can inhibit the decrease of β-carotene in walnut oil, which is indirectly beneficial to the oxidative stability of walnut oil.

**Figure 7:**
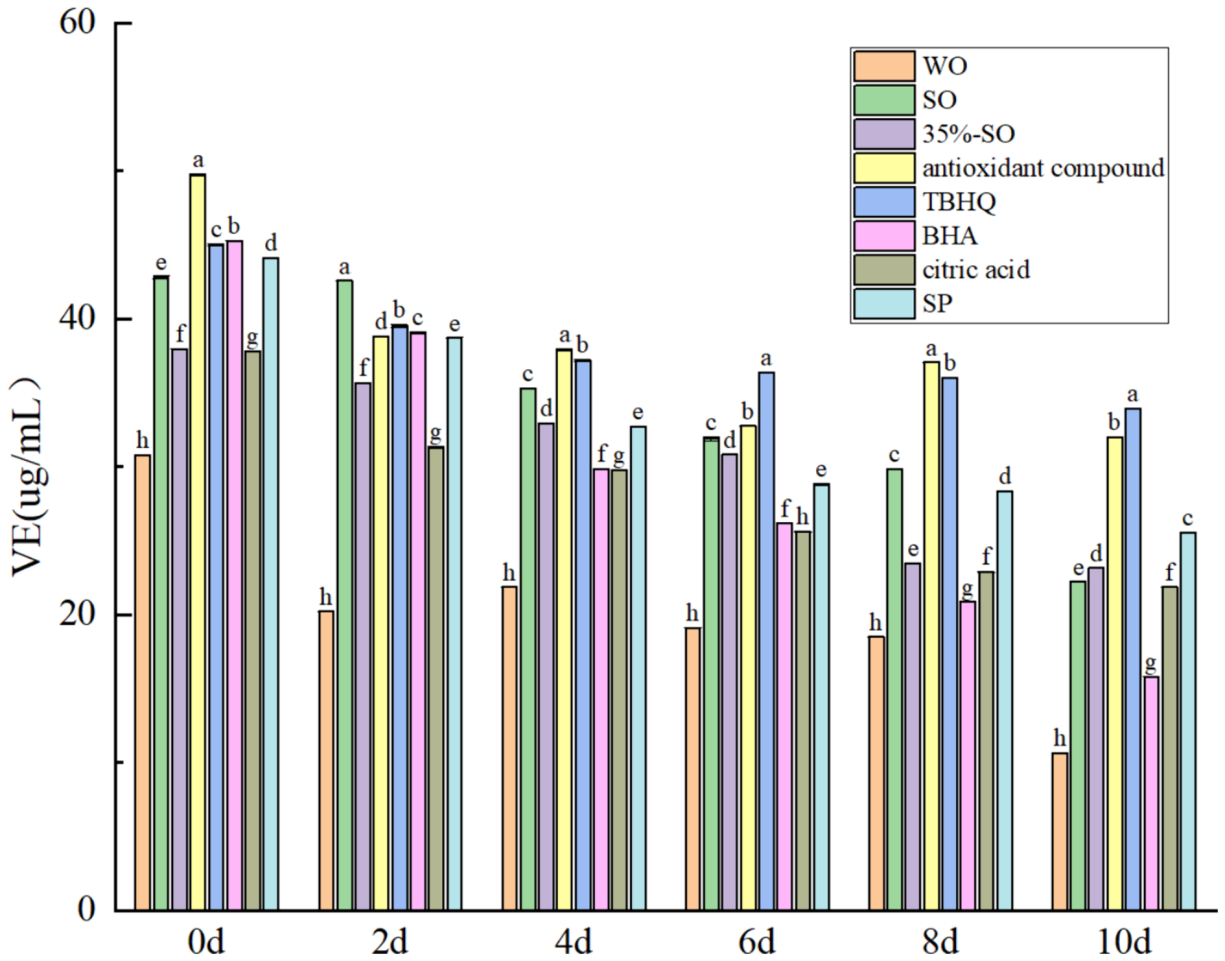
Effects of sesamol on vitamin E in walnut oil.

The vitamin E of all treatment groups decreased gradually with the prolongation of accelerated oxidation time (Figure 7), which was consistent with the results of Ampofo et al. (43), It may be that the content of vitamin E decreases due to oxidation. On the 10th day, the vitamin E content of the blank walnut oil group was the lowest, which was significantly lower than that of the sesame oil group. The loss rate of vitamin E in blank walnut oil was 65.42% while loss rate of vitamin E in the sesame oil was 48.08%, indicating that the vitamin E in the walnut oil was more easily lost. During the oxidation process, vitamin E protected unsaturated fatty acids by competitively binding oxygen with unsaturated fatty acids. The less the loss rate of tocopherol, the more favorable the stability of walnut oil (44). The vitamin E loss rate of the 35%-SO group was 42.08%, and the vitamin E loss rate of the SP group was 38.94%. The loss rate of vitamin E in AC group was 34.04%. The results showed that adding 35%-SO, SP, and AC could reduce the loss of vitamin E in walnut oil, it shows that they can reduce the oxidation of vitamin E, thereby reducing its content. The loss rates of BHA, citric acid, and TBHQ were 65.01, 42.21 and 38.94%, respectively. In summary, several antioxidants can inhibit the decrease of vitamin E in walnut oil, and the inhibitory effect is shown as follows: TBHQ > AC > SP > 35%-SO > sesame oil > citric acid > BHA > walnut oil. The inhibition effect of AC on the loss rate of vitamin E in walnut oil was second only to TBHQ, indicating that AC could have a greater effect on vitamin E in walnut oil than TBHQ, and had a certain protection on vitamin E, which was indirectly beneficial to the stability of walnut oil.

**Figure 8:**
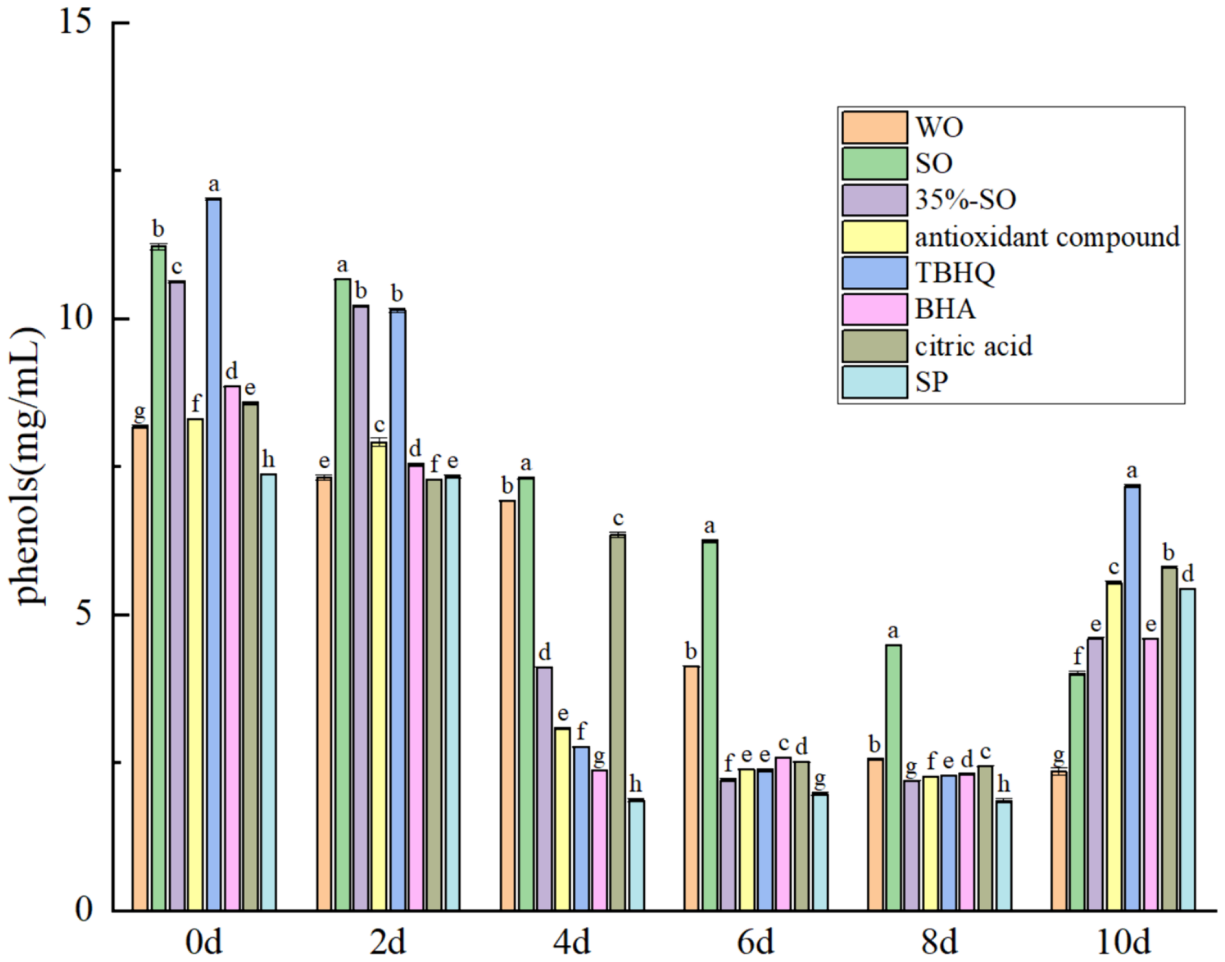
Effects of sesamol on total phenols in walnut oil.

The total phenol content gradually decreased with the extension of accelerated oxidation time (Figure 8). At the 10th day of accelerated oxidation, the phenols content in blank walnut oil was the lowest, with a loss of 71.23%. The total phenol content in sesame oil was significantly higher than that in blank walnut oil (*p < 0.05*), and the loss rate was 64.22%, indicating that phenol in walnut oil were more easily lost. The total phenol loss rates in the 35% sesame oil, AC, and SP groups were 56.63, 33.31, and 26.12%, respectively, indicating that it could better protect the loss of phenol in walnut oil. Among them, SP had the best effect, second only to TBHQ (21.53%). The loss of BHA and citric acid were 51.84 and 42.22%, respectively. In summary, the seven treatment groups can inhibit the decrease of total phenol content in walnut oil, thereby indirectly improving the oxidation stability of walnut oil. The order of the total phenol content reduction is as follows: walnut oil > sesame oil >35%-SO > BHA > citric acid > AC > SP > TBHQ. The loss rate of total phenols in AC and SP was only lower than that of TBHQ, but higher than that of other groups, indicating that AC and SP could protect the polyphenols in walnut oil, which was beneficial to the oxidative stability of walnut oil.

**Figure 9:**
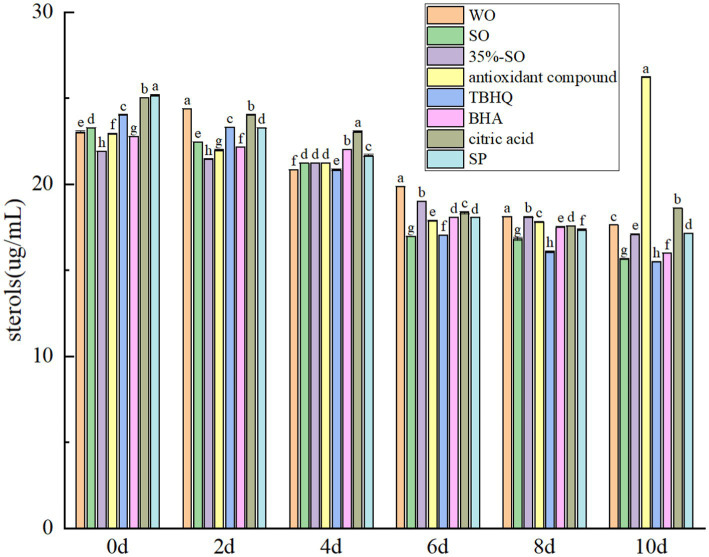
Effects of sesamol on total sterols in walnut oil.

The total sterol content gradually decreased with the extension of accelerated oxidation time (Figure 9). This is because sterols are prone to oxidation reactions and a variety of sterol oxidation products and toxic and harmful substances are derived (45). During the oxidation process, the content of sterols gradually decreases. On the 10th day of accelerated oxidation, the total sterols in blank walnut oil lost 23.45%, and the total sterols in sesame oil lost 37.73%, indicating that the sterols in sesame oil were more easily oxidized. The total sterols in SP and 35% sesame oil lost 31.83 and 21.98%, respectively, whereas the total sterols in AC group did not change significantly, indicating that it could better protect the sterols in walnut oil. It may be due to the synergistic antioxidant effect of citric acid in the compound antioxidant.

**Figure 10:**
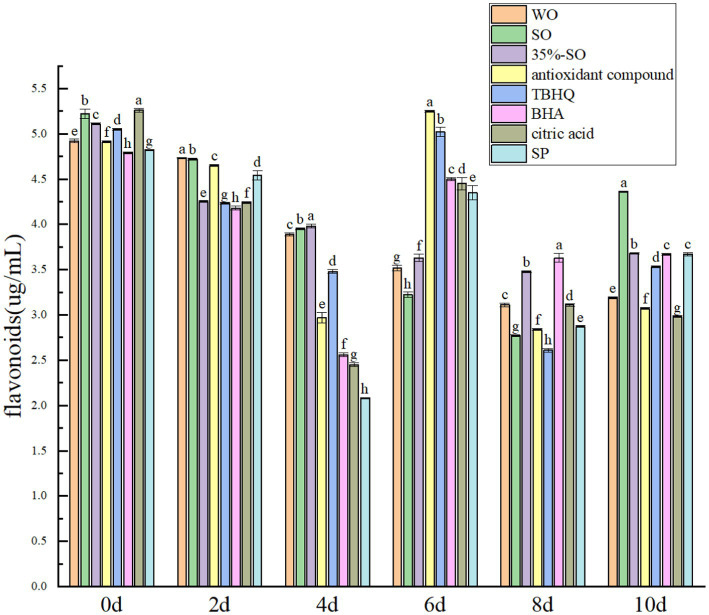
Effects of sesamol on total flavonoids in walnut oil.

**Figure 11:**
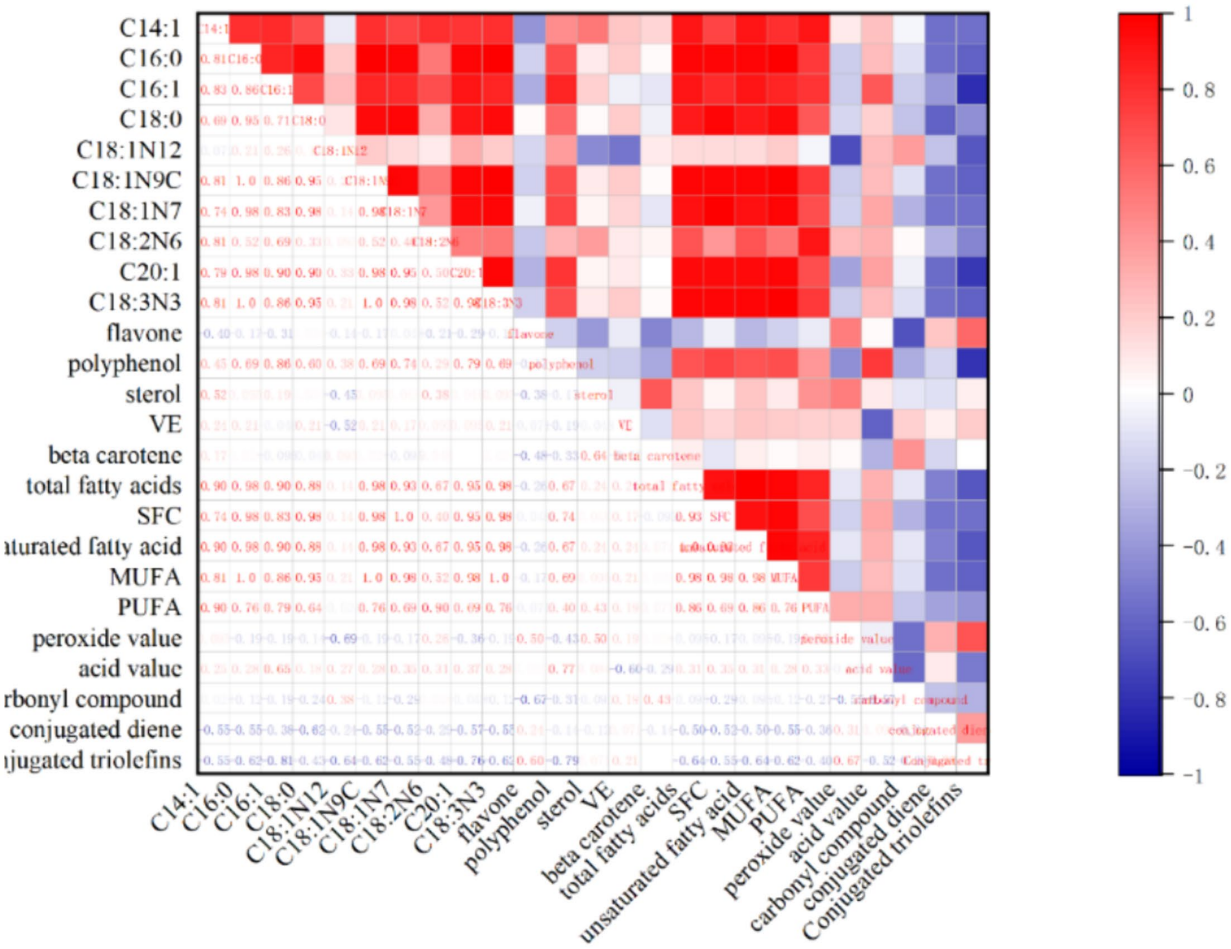
Correlation analysis among endogenous substances.

With the extension of accelerated oxidation time, the total flavonoids in all groups were gradually reduced (Figure 10), which may be due to the destruction of the structure of flavonoids during the accelerated oxidation process, resulting in its reduction. When the oxidation was accelerated to the 10th day, the total flavonoids in the blank walnut oil group lost 36.28%, and the sesame oil group lost 17.07%, indicating that the flavonoids in the walnut oil were more easily oxidized. The total flavonoids of 35%-SO, AC, and SP groups were lost by 28.74, 38.71, and 24.78%, respectively, indicating that SP could better protect the flavonoids in walnut oil. The flavonoid loss rates of TBHQ, BHA, and citric acid groups were 31.03, 24.32, and 44.79%, respectively. The decreasing order of flavonoids in walnut oil was as follows: citric acid > AC > walnut oil > TBHQ >35%-SO > SP > BHA > sesame oil. Flavonoids, as endogenous antioxidants, have certain antioxidant effects, and several antioxidants have different protective effects on flavonoids in walnut oil. Among them, SP shows notable efficacy, indicating that the addition of SP can effectively inhibit the loss of flavonoids in walnut oil, which in turn indirectly contributed to the oxidative stability of walnut oil. Thus, The addition of sesamol and its compound antioxidants can reduce the reduction of polyphenols, flavonoids, sterols, vitamin E, and daucosterol in walnut oil, thereby delaying the oxidative rancidity of walnut oil (Figure 11).

### 3.3 Effects of sesamol on fatty acid content of walnut oil

The total content of 51 fatty acids in walnut oil was 249102.9 ug/mL (Table 2). Main fatty acids were oleic acid, *α*-linolenic acid, palmitic acid, linoleic acid, cis-11-eicosenoic acid, cis-9-myristic acid and stearic acid, and their contents were 38650.84 μg/mL, 31309.77 μg/mL, 23601.32 μg/mL, 119210.3 μg/mL, 10665.49 μg/mL, 8932.27 μg/mL, 6268.71 μg/mL, respectively. At the end of oxidation, monounsaturated fatty acids in walnut oil decreased by 13314.13 ug/mL and polyunsaturated fatty acids decreased by 9238.47 ug/mL. It can be seen that the contents of polyunsaturated fatty acids and monounsaturated fatty acids are decreasing. It may be because unsaturated fatty acids contain more unsaturated bonds and are easily oxidized. The total fatty acid contents of TBHQ, BHA and NMS were 265328.53 ug/mL, 262036.71 ug/mL and 296091.24 ug/mL, respectively. At the end of the accelerated oxidation of fatty acids in walnut oil of TBHQ, the oleic acid content decreased by 6.39%, the linoleic acid content decreased by 1.55%, and the *α*-linolenic acid content decreased by 11.22%. At the end of oxidation, the content of oleic acid, linoleic acid and α-linolenic acid in BHA group decreased by 1.69, 4.28 and 6.68% respectively, and the content of linoleic acid increased by 3.87%. The content of monounsaturated fatty acids and polyunsaturated fatty acids in citric acid group did not decrease.

**Table 2:** Changes of fatty acid content in walnut oil before and after oxidation.

Fatty acids	Time	HO (ug/mL)	ZO (ug/mL)	AC (ug/mL)	35%-SO (ug/mL)	SP (ug/mL)	TBHQ (ug/mL)	BHA (ug/mL)	NMS (ug/mL)
C6:0	Onset	4.9 ± 0.1^a^	8.1 ± 0.4^cd^	4.6 ± 0.7^a^	8.5 ± 3.3^d^	5.5 ± 0.6^b^	5.6 ± 0.6^b^	6.5 ± 1.4^bc^	4.7 ± 1.6^a^
	End	5.3 ± 0.5^a^	8.5 ± 0.4^d^	6.7 ± 0.6^b^	8.2 ± 0.4	7.5 ± 2.5^c^	5.7 ± 0.6^a^	6.6 ± 0.6^b^	7.7 ± 0.2^c^
C8:0	Onset	5.3 ± 0.2^a^	6.2 ± 0.1^b^	5.2 ± 0.2^a^	7.1 ± 2.1^c^	5.6 ± 0.2^a^	5.4 ± 0.1^a^	6 ± 0.5^b^	5 ± 0.4^a^
	End	5.4 ± 0.1^a^	8.3 ± 0.4^c^	6.1 ± 0.2^b^	6.6 ± 0.1^b^	6.7 ± 1.8^b^	5.6 ± 0.4^a^	5.8 ± 0.5^a^	6.3 ± 0.3^b^
C10:0	Onset	3.6 ± 0.1^a^	4.5 ± 0.2^b^	3.4 ± 0.1^a^	4.3 ± 1^b^	3.6 ± 0.1^a^	3.6 ± 0.2^a^	4 ± 0.4^b^	3.4 ± 0.1^a^
	End	3.5 ± 0.1^a^	4.5 ± 0.1^c^	3.7 ± 0.2^b^	3.8 ± 0.1^b^	4.2 ± 1.1^c^	3.5 ± 0.4^a^	3.6 ± 0^a^	3.8 ± 0.1^ab^
C11:0	Onset	6.1 ± 0.1^a^	6.1 ± 0.1^a^	5.9 ± 0.1^a^	7.6 ± 2^c^	6.1 ± 0.2^a^	6.2 ± 0^a^	6.4 ± 0.4^b^	6 ± 0.3^ab^
	End	6.1 ± 0.1^a^	6.2 ± 0.1^a^	6.2 ± 0.1^a^	6.1 ± 0.1^a^	6.7 ± 1.4^b^	5.9 ± 0.2^a^	6.3 ± 0.2^b^	6.3 ± 0.1^b^
C12:0	Onset	6.4 ± 0.2^b^	7.6 ± 0.2^c^	5.8 ± 0.3^a^	7.4 ± 1.3^c^	6.3 ± 0.2^b^	6.8 ± 0.3^b^	6.5 ± 0.9^b^	6.4 ± 0.3^b^
	End	5.8 ± 0.1^a^	7.8 ± 0.6^c^	6.7 ± 0.5^b^	6.7 ± 0.2^b^	7.1 ± 0.8^b^	6.2 ± 0.2^a^	6.4 ± 0.4^ab^	6.9 ± 0.3^b^
C13:0	Onset	6.5 ± 0.1^a^	6.1 ± 0.2^a^	6.2 ± 0.1^a^	7.8 ± 2.1^b^	6.3 ± 0.1^a^	6.5 ± 0.2^a^	6.7 ± 0.4^a^	6.4 ± 0.4^a^
	End	6.3 ± 0.1^a^	6.3 ± 0.1^a^	6.4 ± 0.3^a^	6.3 ± 0.2^a^	7.3 ± 1.6^b^	6.3 ± 0.2^a^	6.7 ± 0.2^b^	6.6 ± 0.3^a^
C14:0	Onset	60.4 ± 1.8^c^	58.3 ± 3.8^b^	56.8 ± 2.1^b^	53.1 ± 2^a^	56.9 ± 3.3^b^	62.5 ± 4.8^c^	60.7 ± 3.6^c^	67.3 ± 10.3^d^
	End	50.5 ± 3.8^a^	60.4 ± 4.9^d^	63.2 ± 5.8^e^	54.1 ± 1.7^b^	64.2 ± 2.9^e^	58.1 ± 4.5^c^	58.2 ± 9.4^c^	76.1 ± 2.3^f^
C14:1 T	Onset	514.2 ± 45.2^c^	519.4 ± 98.5^d^	449.8 ± 55.5^a^	508.4 ± 4.6^b^	529.9 ± 42.3^f^	556.2 ± 55.5^g^	523.7 ± 12.9^e^	561.6 ± 7 h
	End	392.3 ± 40.8^a^	586.3 ± 66.2^e^	582.9 ± 100.8^e^	492.3 ± 33.3^b^	517.1 ± 53.1^d^	585.2 ± 27.6^e^	584 ± 21.1^e^	508.1 ± 35.6^c^
C14:1	Onset	8932.3 ± 663.2^b^	8883.3 ± 2285.4^b^	6812.4 ± 2210.1^ab^	8321.7 ± 2467.4^b^	8192.5 ± 1891.2^b^	8344.6 ± 2015.4^b^	4515.4 ± 2281.9^a^	4251.4 ± 1482.9^a^
	End	5669.8 ± 204.7^ab^	10506.7 ± 1057.8^c^	9828.3 ± 2391.6^bc^	8719.2 ± 777.1^b^	7885.9 ± 2138^b^	6850.5 ± 2130.5^b^	3505.6 ± 1638.9^a^	4856.6 ± 743.9^ab^
C15:0	Onset	68.5 ± 2.1^cd^	20.1 ± 0.5^a^	62.3 ± 1.4^cd^	47 ± 1^b^	61.5 ± 3.9^cd^	70 ± 4.7^cd^	66.9 ± 3.3^cd^	74.2 ± 11.9^d^
	End	56.8 ± 5.1^cd^	20.4 ± 0.8^a^	68.9 ± 6.2^cd^	47.3 ± 1.5^b^	70.8 ± 1.8^cd^	64.7 ± 4.8^cd^	64.8 ± 9.1^cd^	82.7 ± 2.9^d^
C15:1 T	Onset	101.2 ± 12.3^a^	120.8 ± 1^ab^	132 ± 16.8^bc^	153.1 ± 13.4^cd^	142.7 ± 1.9^b^	149 ± 7.4^c^	174 ± 22.2^d^	193.4 ± 7.8^f^
	End	118.3 ± 5.9^a^	185.9 ± 48.3^bc^	166.7 ± 26.7^b^	137.9 ± 26.6^b^	143.1 ± 15.3^b^	196.6 ± 43^bc^	225.5 ± 29.3^c^	155.8 ± 22.4^b^
C15:1	Onset	135.6 ± 11.3^ab^	138.8 ± 18.6^ab^	131 ± 13.3^a^	142.9 ± 5.3^ab^	147.9 ± 9.3^ab^	153.8 ± 13.6^bc^	149.7 ± 4^ab^	163 ± 4.6^d^
	End	111.5 ± 6.9^a^	163.1 ± 19.6^b^	161.2 ± 22.5^b^	139.7 ± 10.1^b^	142 ± 15.7^b^	169.3 ± 13.6^b^	167.3 ± 12.9^b^	146.9 ± 11.7^b^
C16:0	Onset	23601.3 ± 744.1^ab^	28142.3 ± 1477.4^c^	21344.2 ± 950.4^a^	20496.5 ± 2224.4^a^	20567.3 ± 1806.1^a^	23875.2 ± 2132.8^ab^	22953.6 ± 1435.2^a^	26136.9 ± 5291.9^bc^
	End	18505.8 ± 2033.1^ab^	28469.7 ± 2333.3^c^	23696.2 ± 2621^ab^	21917.6 ± 1312.7^a^	24561.2 ± 788.4^ab^	21861.3 ± 2010.7^a^	21620.4 ± 4564.6^a^	30205.7 ± 1039.4^c^
C16:1 T	Onset	333.2 ± 43.1^ab^	347.6 ± 57.3^ab^	307.8 ± 35.9^a^	345.5 ± 5.9^ab^	361.1 ± 30.4^b^	380.2 ± 34.1^b^	362.4 ± 5.1^ab^	391.6 ± 6.9^b^
	End	272.6 ± 24.9^a^	405.1 ± 44.8^b^	394.7 ± 64.8^b^	342.5 ± 21.4^ab^	351.3 ± 42.6^ab^	398.3 ± 19.6^b^	397.6 ± 15.8^b^	353.1 ± 26.1^ab^
C16:1	Onset	1103.2 ± 63.6^c^	813.5 ± 97.3^a^	1022.6 ± 43.7^bc^	919.5 ± 48.8^ab^	1080.1 ± 91.5^bc^	1181.3 ± 101.8^c^	1133.9 ± 51.7^cd^	1275.9 ± 155.8^d^
	End	883.1 ± 88.5^a^	883 ± 74.1^a^	1210.6 ± 148.3^ab^	945.2 ± 2.6^a^	1158.8 ± 55.1^ab^	1133.8 ± 74.7^ab^	1162.2 ± 119.6^ab^	1364.2 ± 35.3^b^
C17:0	Onset	166.7 ± 4.8^bc^	138.4 ± 6.6^ab^	153.7 ± 2.1^ab^	133.3 ± 7.3^a^	148.1 ± 12.8^ab^	169.6 ± 12.5^bc^	163.5 ± 9.1^bcd^	182.1 ± 31.7^d^
	End	137.3 ± 13.5^a^	140.8 ± 11.5^a^	167.2 ± 17.6^b^	138.8 ± 6.2^a^	173.7 ± 4.6	156.4 ± 11.3^ab^	155.6 ± 23.7^ab^	203.2 ± 5.8^c^
C17:1 T	Onset	163.6 ± 9.4^a^	160.1 ± 27.2^a^	141.1 ± 16.3^a^	157.5 ± 15.9^a^	162.6 ± 11.3^a^	165.1 ± 14.3^a^	145 ± 0.7^a^	155.9 ± 13.2^a^
	End	126.1 ± 7.4^a^	184 ± 14.8^c^	174.7 ± 18.1^b^	159.8 ± 9^b^	156.4 ± 22.9^ab^	172.9 ± 13.5^bc^	147.9 ± 13.7^ab^	144.6 ± 11.5^ab^
C17:1	Onset	261.1 ± 14.2^a^	235.5 ± 29.8^a^	234.2 ± 17.9^a^	233.2 ± 31.4^a^	252 ± 14.3^a^	257.7 ± 26.7^a^	224.6 ± 22.9^a^	237.3 ± 32.4^a^
	End	208.3 ± 13.1^a^	266.8 ± 18.6^bc^	275.8 ± 22^c^	244.8 ± 7.7^b^	255.8 ± 32.2^b^	265.4 ± 30.8^bc^	200.1 ± 21.5^a^	248.3 ± 23.2^b^
C18:0	Onset	6268.7 ± 174.6^ab^	15,719 ± 802.8^d^	5529.6 ± 115.3^ab^	7438.2 ± 787^c^	5290.3 ± 548.4^a^	6147.5 ± 583.5^abc^	5892.4 ± 370.3^ab^	6779.7 ± 1445.5^abc^
	End	4882.6 ± 584.8^a^	16149.3 ± 1486.9^e^	6014.2 ± 678.4^b^	8078.4 ± 379.6^d^	6539.1 ± 101.8^b^	5,600 ± 439.2^a^	5619.2 ± 1072.6^a^	7695.3 ± 244.2^c^
C18:1 N12T	Onset	113.5 ± 6.1^a^	126.2 ± 21.2^ab^	110.1 ± 6.5^a^	125.5 ± 2.1^ab^	130.3 ± 14.5^ab^	134.6 ± 14.2^ab^	139.3 ± 20.1^ab^	153 ± 22.2^b^
	End	93.5 ± 6.4^a^	141.2 ± 14.4^b^	135.6 ± 30.9^ab^	124 ± 7.6^ab^	125.7 ± 4^ab^	138.9 ± 14^ab^	164.7 ± 15.2^b^	140 ± 20.7^ab^
C18:1 N9T	Onset	194.7 ± 6.2^a^	230.3 ± 29^ab^	213.5 ± 8.5^a^	229.8 ± 16^ab^	244.4 ± 26.3^abc^	267.9 ± 19.2^c^	291.5 ± 41.4^c^	322.6 ± 49.2^d^
	End	179.7 ± 21.2^a^	248 ± 20.3^b^	249.5 ± 59.5^b^	227.2 ± 11.8^b^	247.7 ± 4^b^	268.4 ± 17.3^b^	335.3 ± 10.6^c^	279.7 ± 33.2^bc^
C18:1 N7T	Onset	315.1 ± 28.9^a^	346.1 ± 47.3^a^	288.4 ± 29.5^a^	320 ± 7.4^a^	334.3 ± 35.7^a^	342.8 ± 34.4^a^	306.9 ± 23.7^a^	311.2 ± 45.5^a^
	End	256.4 ± 18.4^a^	378.1 ± 34.9^c^	361.8 ± 58.9^c^	321.6 ± 18.1^bc^	326.7 ± 35.9^bc^	351.2 ± 12.6^bc^	301.7 ± 25.8^b^	314 ± 10.8^b^
C18:1 N12	Onset	1265.9 ± 208.5^a^	2303.1 ± 681.4^a^	2800.5 ± 1544.9^ab^	2671.2 ± 2367.5^ab^	2669.8 ± 1603.3^ab^	2889.4 ± 887.7^ab^	3,489 ± 1635.1^ab^	5322.3 ± 812.7^b^
	End	1320.9 ± 64.4^a^	993.3 ± 212.5^a^	2091.1 ± 1213.9^ab^	1,191 ± 147.8^a^	3090.3 ± 383.5^b^	2453.7 ± 817.7^ab^	5113.4 ± 1140.9^c^	7258.4 ± 3381.1^d^
C18:1 N9c	Onset	38650.8 ± 1942.9^a^	80728.6 ± 574.8^c^	36216.1 ± 1412.7^a^	46504.3 ± 4343.8^b^	34740.7 ± 2217.8^a^	40229.4 ± 3155.7^ab^	40188.4 ± 2102.1^ab^	46478.5 ± 8498.4^b^
	End	32074.9 ± 3529.8^a^	78498.3 ± 3009.2^c^	39953.2 ± 3527.3^a^	49146.6 ± 2400.8^b^	40849.1 ± 486.8^a^	37811.9 ± 3787.7^ab^	39509.1 ± 6182.9^ab^	53486.7 ± 2593.5^b^
C18:1 N7	Onset	3706.4 ± 195.3^ab^	5156.5 ± 2883.5^b^	3346.4 ± 80.6^ab^	2916.4 ± 272^a^	3326.7 ± 275.8^ab^	3842.9 ± 410^ab^	3723.4 ± 215.6^ab^	4353.1 ± 907.9^ab^
	End	2941.7 ± 392.5^a^	5214.3 ± 2677.8^b^	3850.6 ± 403.5^ab^	3133.3 ± 137^a^	3963.8 ± 159.2^ab^	3502.4 ± 313.3^ab^	3564.2 ± 646.7^ab^	4886.5 ± 211.9^ab^
C18:2 N6T	Onset	96.6 ± 8.2^ab^	108.5 ± 13.5^b^	87.4 ± 4.8^a^	102.5 ± 1.2^b^	103 ± 6.6^b^	105.1 ± 9.2^b^	101.1 ± 0.8^b^	110.4 ± 4.7^b^
	End	84.6 ± 3.8^a^	116.7 ± 8^b^	107.2 ± 16.4^b^	99.9 ± 5^ab^	99.6 ± 6.3^ab^	106.7 ± 6.1^b^	108.8 ± 6.8^b^	105.7 ± 7.8^b^
C19:1 N12T	Onset	110.2 ± 9.8^a^	130 ± 8.2^ab^	112.1 ± 1.2^a^	113.3 ± 3.8^a^	122.4 ± 35.4^a^	121.8 ± 24.1^ab^	110.3 ± 11.5^a^	101.7 ± 14.5^a^
	End	104.6 ± 5^ab^	151 ± 24.6^c^	123.8 ± 40.4^b^	105 ± 12.9^ab^	102 ± 17.3^a^	140.7 ± 5.8^c^	99.3 ± 16.2^a^	91.4 ± 18.9^a^
C19:1 N9T	Onset	46.1 ± 2.8^ab^	60.9 ± 3^b^	47.9 ± 1.9^ab^	49.9 ± 4.4^a^	47.7 ± 11.9^abb^	45.9 ± 5.7^ab^	47.6 ± 6.2^ab^	43.4 ± 6.6^a^
	End	44.7 ± 6.5^a^	62.2 ± 8.3^b^	47.9 ± 10.1^a^	45 ± 7.2^a^	43.6 ± 8.2^a^	55.8 ± 3.4^ab^	43 ± 5.1^a^	40.7 ± 7.5^a^
C18:2 N6	Onset	119210.3 ± 3385.5^ab^	110,880 ± 4524.4^a^	122,005 ± 1630.2^bc^	113700.2 ± 10086.5^abc^	125,209 ± 5374.4^b^	130993.5 ± 6867.1^b^	133367.3 ± 4762.8^c^	146724.8 ± 12270.7^d^
	End	116956.6 ± 8696^a^	110735.8 ± 6984.1^a^	132690.2 ± 5172.9^bc^	118632.3 ± 3065^abc^	123147.8 ± 1529.2^b^	128985.4 ± 10929.2^b^	138523.9 ± 9796^c^	154891.7 ± 2179.4^d^
C20:0	Onset	228 ± 13^a^	1795.7 ± 111.9^c^	206.2 ± 10.9^a^	586.7 ± 59.7^b^	199.5 ± 17.6^a^	227.2 ± 20.8^a^	225 ± 11.9^a^	266.5 ± 52.5^a^
	End	181.8 ± 24.8^a^	1776.4 ± 136.6^c^	223.1 ± 22.7^a^	633.3 ± 27.5^b^	237.8 ± 7.7^a^	215 ± 20.6^a^	221.3 ± 40.3^a^	299.2 ± 5.9^a^
C18:3 N6	Onset	61.5 ± 5.6^a^	58.2 ± 1.8^a^	64.4 ± 8.1^a^	59.2 ± 2.6^a^	65.6 ± 5.6^a^	86 ± 7.1^ab^	88.7 ± 10.7^ab^	123.1 ± 19.5^c^
	End	57.4 ± 4.3^a^	59.8 ± 3.8^a^	75.6 ± 18.1^ab^	54.1 ± 1.1^a^	78.8 ± 6.2^ab^	88.9 ± 17.7^b^	101.6 ± 16.1^bc^	105.2 ± 19.6^bc^
C20:1 T	Onset	117.7 ± 11.9^a^	119.8 ± 15.8^a^	118.5 ± 5.7^a^	121.3 ± 8.5^a^	135.8 ± 5.5^ab^	152.9 ± 12.1^b^	171.2 ± 24.1^b^	240.7 ± 13.2^c^
	End	104.2 ± 9.5^a^	133 ± 8.3^ab^	151.5 ± 29.5^ab^	118.6 ± 4.4^a^	133.2 ± 8.1^ab^	173.5 ± 17.7^b^	215.8 ± 1.2^c^	191.2 ± 35.3^b^
C20:1	Onset	10665.5 ± 434.4^c^	558.3 ± 41.1^a^	9917.4 ± 309.4^c^	6136.9 ± 401^b^	9658.5 ± 955.2^c^	11257.7 ± 1216.6^cd^	11064.4 ± 837.9^cd^	12862.8 ± 2599.1^d^
	End	8620.9 ± 1012.9^c^	582.8 ± 44.3^a^	11094.5 ± 1176.7^c^	6477.3 ± 396.4^b^	11582.5 ± 169.6^c^	10251.2 ± 1121.5^cd^	10520.7 ± 2042^cd^	14972.7 ± 560.1^d^
C18:3 N3	Onset	31309.8 ± 1287.2^ab^	909.3 ± 70.4^a^	27794.9 ± 1381.2^c^	17155.8 ± 2132.5^b^	27842.5 ± 2208.1^c^	31864.6 ± 2850.7^cd^	31173.5 ± 1753.6^cd^	36790.3 ± 7592.1^e^
	End	24389.4 ± 3698.1^b^	905.5 ± 76.4^a^	31606.4 ± 2531.9^d^	18527.2 ± 1266.1^b^	32815.1 ± 1210.8^d^	28650.4 ± 2935.8^c^	29088.7 ± 6196.3^c^	42728.7 ± 2027.4^e^
C21:0	Onset	42.1 ± 1.2^a^	29.1 ± 1.5^a^	37.6 ± 0.6^a^	34.1 ± 1^a^	36.4 ± 1.9^a^	41.2 ± 3.1^a^	39.8 ± 1.8^a^	44.8 ± 7.4^a^
	End	35.3 ± 2.8^a^	28.9 ± 1.7^a^	39.8 ± 3.2^a^	34 ± 1.7^a^	43.4 ± 2.4^ab^	37.6 ± 2.4^a^	37.8 ± 4.6^a^	50.1 ± 0.2^b^
C20:2	Onset	126.4 ± 9.9^a^	127 ± 8.2^a^	113.5 ± 3.3^a^	121.2 ± 8.4^a^	124.2 ± 8^a^	133.3 ± 12.5^a^	126.1 ± 2^a^	153.1 ± 23.1^b^
	End	109.6 ± 8.4^a^	131.9 ± 6.8^ab^	133.6 ± 12.3^ab^	117.3 ± 3.3^a^	128 ± 1.2^ab^	129.7 ± 14.2^ab^	132.9 ± 4.6^ab^	160.7 ± 7.9^c^
C22:0	Onset	67.7 ± 4.1^a^	318.9 ± 15^c^	62.1 ± 1.1^a^	120.3 ± 9.7^b^	59.4 ± 4.2^a^	67.1 ± 5.7^a^	65.7 ± 3.4^a^	73.7 ± 12.9^a^
	End	56 ± 6.4^a^	324.9 ± 26.7^c^	65.6 ± 4.9^a^	126.8 ± 6.6^b^	73.5 ± 1.5^a^	63.6 ± 5.1^a^	63.2 ± 8.6^a^	84.5 ± 1.2^a^
C20:3 N6	Onset	96 ± 7.3^a^	102.4 ± 13.3^a^	100.3 ± 11.6^a^	113.3 ± 16.6^ab^	115.8 ± 10.5^ab^	121.8 ± 13^b^	109.7 ± 8.1^ab^	110.4 ± 1.9^ab^
	End	86.4 ± 6.4^a^	113 ± 12^a^	131.9 ± 21^b^	106.6 ± 4.7^a^	111.9 ± 5.7^a^	120.8 ± 2^ab^	126.6 ± 16.1^ab^	120.7 ± 2.2^ab^
C22:1 N9T	Onset	134.7 ± 12^b^	131.7 ± 19.9^b^	114.9 ± 9.4^a^	132.4 ± 18.1^b^	135.7 ± 7.2^b^	145.7 ± 22.7^b^	167.4 ± 20.8^b^	178.4 ± 22.3^c^
	End	112.6 ± 11.3^a^	151.1 ± 11.1^b^	149 ± 21^b^	127.8 ± 2.7^a^	127.5 ± 14.6^a^	150.3 ± 13.5^b^	198.5 ± 31.3^c^	177.8 ± 15.7^bc^
C22:1 N9	Onset	85.1 ± 10.2^b^	81.6 ± 15.9^b^	60.7 ± 7.1^a^	83 ± 14.9^b^	81.8 ± 5.1^b^	79.2 ± 14.3^b^	61.9 ± 7.9^a^	82.6 ± 11^b^
	End	67.8 ± 10.2^a^	99.7 ± 8.2^c^	82 ± 14^b^	74.6 ± 2.4^a^	69.6 ± 11.2^a^	76.6 ± 16.1^a^	75.5 ± 9.8^a^	91.6 ± 6.2^c^
C20:3 N3	Onset	22.9 ± 2.7^a^	20.8 ± 2.8^a^	18.1 ± 0.9^a^	25.7 ± 0.9^a^	21.9 ± 1.8^a^	23.1 ± 3.7^a^	21.8 ± 0.3^a^	24.7 ± 3^a^
	End	22.3 ± 2^a^	21.8 ± 0.7^a^	25.1 ± 3.2^a^	20.3 ± 0.9^a^	23.1 ± 2.1^a^	21.8 ± 5.6^a^	23.8 ± 3.7^a^	30.2 ± 0.8^b^
C20:4 N6	Onset	35.4 ± 6.4^b^	32.7 ± 3.2^b^	24.9 ± 1.9^a^	40.6 ± 1.2^c^	33.6 ± 5.2^b^	32.1 ± 5.6^ab^	29.1 ± 1.7^ab^	35.9 ± 5.9^b^
	End	34.1 ± 3.3^a^	35.5 ± 1^a^	33.7 ± 3.9^a^	30.5 ± 3.2^a^	30.1 ± 2.4^a^	29.5 ± 10.3^a^	33.1 ± 6.6^a^	41.6 ± 2.7^ab^
C23:0	Onset	30.6 ± 0.6^a^	58.2 ± 2.3^c^	28.6 ± 0.7^a^	37.1 ± 1.4^b^	28 ± 1.6^a^	30.3 ± 3^a^	30.4 ± 1.4^a^	31.6 ± 4^a^
	End	27.5 ± 1^a^	57.7 ± 3.7^c^	30.5 ± 2.1^a^	35.8 ± 1.4^ab^	33 ± 2.4^ab^	28.2 ± 1.9^a^	29 ± 2.4^a^	36.1 ± 1.2^ab^
C22:2	Onset	48.7 ± 6^a^	48.2 ± 5.6^a^	44.4 ± 0.7^a^	49.5 ± 7.6^a^	52.5 ± 5^ab^	55.6 ± 6.5^ab^	52.9 ± 2.7^ab^	63.8 ± 4.6^b^
	End	46.4 ± 4.4^a^	53.9 ± 1.4^ab^	55.2 ± 4.7^ab^	46.8 ± 1.8^a^	46.8 ± 3.8^a^	54.9 ± 7.9^ab^	65 ± 3^b^	63.8 ± 6.2^b^
C20:5 N3	Onset	26.7 ± 2.9^a^	22.9 ± 2.2^a^	19.5 ± 0.8^a^	29.9 ± 6.6^a^	24 ± 2.7^a^	24.3 ± 3.2^a^	30.4 ± 14.4^a^	27.7 ± 4.5^a^
	End	23.7 ± 2.9^a^	24.1 ± 1.1^a^	24.1 ± 2.5^a^	22.6 ± 1.4^a^	28.5 ± 10.8^ab^	23.7 ± 5.3^a^	24.7 ± 4.1^a^	27.2 ± 2.6^ab^
C24:0	Onset	27 ± 1.3^a^	174.9 ± 7.4^c^	24 ± 0.4^a^	61.5 ± 6.1^b^	23.5 ± 1.4^a^	25.8 ± 2^a^	25.5 ± 2.1^a^	26.7 ± 3.8^a^
	End	22.5 ± 1.9^a^	179.6 ± 11.2^c^	25.6 ± 2.1^a^	65.8 ± 3.4^b^	27.8 ± 0.8^a^	24.2 ± 2^a^	24.6 ± 1.8^a^	30.2 ± 1.1^a^
C24:1	Onset	407.7 ± 62.3^c^	394.8 ± 54.9^bc^	288.5 ± 13.6^a^	358.1 ± 91.7^bc^	381 ± 10.7^bc^	363.6 ± 58.8^bc^	323.8 ± 49.9^b^	432.5 ± 35.6^c^
	End	339 ± 54^a^	460.5 ± 27.7^c^	370 ± 45.9^a^	394.6 ± 10.9^b^	328.4 ± 81.9^a^	402.7 ± 45^bc^	368.9 ± 39.2^b^	392.5 ± 36.1^b^
C22:4	Onset	33 ± 4.6^c^	22.7 ± 2.6^ab^	14.3 ± 1.2^a^	23.7 ± 2.5^ab^	22.9 ± 1.7^ab^	21.7 ± 6.2^ab^	18.4 ± 0.9^a^	27.9 ± 5.3^ab^
	End	24.8 ± 6.7^b^	25.5 ± 1.7^b^	23.3 ± 4.3^b^	20.4 ± 1.8^a^	18.2 ± 1.9^a^	19.4 ± 6^a^	23.2 ± 6.8^b^	38.9 ± 26.6^c^
C22:5 N6	Onset	29.3 ± 5.6^c^	20.8 ± 3^b^	10.6 ± 1.1^a^	23.4 ± 3.8^b^	24.2 ± 6.3^b^	18.7 ± 5.7^ab^	15.1 ± 0.9^ab^	24.5 ± 4.2^bc^
	End	23.6 ± 6^b^	23.2 ± 1.2^b^	19.5 ± 4^ab^	17.4 ± 2.3^a^	13.7 ± 1.5^a^	16.4 ± 6.4^a^	20.4 ± 8.4^ab^	24.7 ± 3.2^b^
C22:5 N3	Onset	24.7 ± 8.1^c^	18 ± 1.7^ab^	8.5 ± 0.2^a^	21 ± 5.1^b^	17.7 ± 3.5^ab^	15 ± 4.9^ab^	12.1 ± 1.7^a^	19.5 ± 3.1^ab^
	End	23 ± 7^b^	21.4 ± 2.3^b^	15.7 ± 3.8^a^	14.7 ± 2.2^a^	12.4 ± 1.8^a^	14.9 ± 5.4^a^	16.8 ± 6.5^a^	22.1 ± 3.8^b^
C22:6 N3	Onset	29.6 ± 11.9^c^	23.2 ± 4.7^ab^	12.3 ± 1.2^a^	27.6 ± 5.6^bc^	21.4 ± 3.4^b^	21.5 ± 7.5^b^	17.1 ± 2.2^a^	27.2 ± 2.5^bc^
	End	30.4 ± 9.2^bc^	28 ± 2^b^	26.5 ± 5.3^b^	22 ± 2.8^ab^	19.6 ± 0.7^a^	21.4 ± 7.3^a^	26.5 ± 9.3^ab^	32.2 ± 4.2^c^

The results are presented mean ± SD (*n* = 3). Different letters indicated that the difference between the groups was statistically significant (*p* < 0.05).

The total fatty acid content in walnut oil added with SP was 243060.67 ug/mL, and the content at the end of oxidation increased by 6.98%. At the end of oxidation, the main monounsaturated fatty acids of cis-9-palmitic acid, apietic acid, oleic acid, isooleic acid and cis-11-eicosenoic acid in walnut oil added with SP decreased by 6.79, 13.61, 14.95 and 16.61%, respectively. The polyunsaturated fatty acid linoleic acid increased by 1.67% and *α*-linolenic acid decreased by 15.15%, respectively. The total fatty acid content of 35%-SO was 231087.61 ug/mL. The content of saturated fatty acids in walnut oil added with 35%-SO increased by 7.29%, and the content of monounsaturated fatty acids and polyunsaturated fatty acids did not decrease, indicating that the addition of 35%-SO could inhibit the oxidation of unsaturated fatty acids in walnut oil, thereby improving the oxidation stability of walnut oil. After adding AC, the total fatty acid content was 240720.57 ug/mL. After the end of oxidation, the content of linoleic acid and *α*-linolenic acid in walnut oil added with AC increased by 8.76 and 13.71%, respectively. The content of monounsaturated fatty acid oleic acid, cis-11-eicosenoic acid and cis-9-myristic acid increased by 10.31, 11.86 and 44.26%, respectively. The main monounsaturated fatty acid oleic acid, cis-9-myristic acid, cis-9-palmitic acid and isooleic acid in blank walnut oil decreased by 20.50, 57.54, 24.92 and 25.99%, respectively. The content of polyunsaturated fatty acids linoleic acid and *α*-linolenic acid decreased by 1.92 and 28.37%, respectively. It shows that the addition of several antioxidants can inhibit the change of unsaturated fatty acid content in walnut oil, and the content of unsaturated fatty acids in SP and AC groups is increasing, indicating that it can improve the quality of walnut oil and prolong the oxidation stability of walnut oil.

Walnut oil is rich in unsaturated fatty acids. The main unsaturated fatty acids are cis-9-myristic acid, oleic acid, isooleic acid, linoleic acid, cis-11-eicosenoic acid, *α*-linolenic acid, and their contents are 3.59, 15.52, 1.49, 47.86, 4.28, and 12.57%, respectively (Table 3). The main saturated fatty acids are palmitic acid and stearic acid, and their contents are 9.47 and 2.52%, respectively. The more unsaturated fatty acids in the oil, the easier it is to be oxidized (46). Unsaturated fatty acids in walnut oil are mainly polyunsaturated fatty acids, while polyunsaturated fatty acids are easily oxidized. At the end of oxidation, the contents of saturated fatty acids, unsaturated fatty acids, monounsaturated fatty acids, and polyunsaturated fatty acids in walnut oil decreased by 21.59, 10.31, 19.77, and 6.11%, respectively, whereas the contents of oleic acid, linoleic acid, and α-linolenic acid in walnut oil decreased by 20.50, 1.92, and 28.37%, respectively, indicating that the unsaturated fatty acids in walnut oil were oxidized and the content decreased. The content of saturated fatty acids in sesame oil increased by 1.62%, unsaturated fatty acids decreased by 0.65%, and the contents of monounsaturated fatty acids and polyunsaturated fatty acids decreased by 1.27 and 0.09%, respectively, which were significantly lower than those in walnut oil (*p* < 0.05). The content of unsaturated fatty acids in sesame oil was lower than that in walnut oil, indicating that the oxidation stability of sesame oil was greater than that of walnut oil.

**Table 3:** Effects of different antioxidants on the content of fatty acids in walnut oil.

Samples	UFA(μg/mL)	SFC (μg/mL)	MUFA (μg/mL)	PUFA (μg/mL)	Proportion of unsaturated fatty acid (%)	Proportion of saturated fatty acid (%)
HO	218508.9 ± 7504^a^	30593.93 ± 939^c^	67358.01 ± 2828^d^	151150.9 ± 4676^a^	87.72 ± 3.01^b^	12.28 ± 0.37^c^
HE	195956.3 ± 17645^d^	23988.93 ± 2670^d^	54043.89 ± 5240^c^	141912.5 ± 12419^b^	89.09 ± 8.72^a^	10.91 ± 1.21^d^
ZO	213981.8 ± 8506^b^	46494.02 ± 2407^b^	101586.9 ± 4705^a^	112394.9 ± 4604^c^	82.15 ± 3.26^c^	17.84 ± 1.21^b^
ZE	212590.4 ± 12334^c^	47249.9 ± 4001^a^	100294.3 ± 5366^b^	112296.1 ± 7067^d^	81.81 ± 4.76^d^	18.18 ± 0.92^a^
HO-TBHQ	234,578 ± 14659^d^	30750.57 ± 2772^c^	71061.61 ± 6621^c^	163516.4 ± 8491^e^	88.41 ± 525^e^	11.59 ± 1.04^b^
HE-TBHQ	223833.1 ± 18710^f^	28142.36 ± 2550^e^	65549.23 ± 5099^f^	158283.9 ± 13620^f^	88.83 ± 7.42^b^	11.17 ± 0.99^e^
HO-BHA	232,477 ± 6272^e^	29559.7 ± 1839^d^	67313.77 ± 3887^d^	165163.2 ± 4476^d^	88.72 ± 2.39^c^	11.28 ± 0.7^d^
HE-BHA	235216.3 ± 22006^c^	27929.52 ± 5730^f^	66900.35 ± 11557^e^	168315.9 ± 12632^c^	89.39 ± 8.36^a^	10.61 ± 2.17^f^
HO-NMS	262375.8 ± 30657^b^	33715.43 ± 6870^b^	78112.6 ± 13241^b^	184263.2 ± 18462^b^	88.61 ± 10.35^d^	11.37 ± 2.32^c^
HE-NMS	288494.2 ± 4390^a^	38800.61a ± 1293^a^	90100.82 ± 535^a^	198393.4 ± 3857^a^	88.14 ± 1.34^f^	11.85 ± 0.39^a^
HO-35%	202037.2 ± 17540^b^	29050.36^b^ ± 3078^a^	70543.88 ± 5406^b^	131493.4 ± 12260^b^	87.43 ± 7.59^a^	12.57 ± 1.33^b^
HE-35%	210400.4 ± 6474^a^	31169.53 ± 1742^a^	72668.14 ± 2270^a^	137732.3 ± 4230^a^	87.10 ± 2.68^b^	12.90 ± 0.72^a^
HO-AC	213184.3 ± 2568^b^	27536.32 ± 1081^b^	62866.14 ± 2225^b^	150318.1 ± 2316^b^	88.56 ± 1.67^b^	11.44 ± 0.44^a^
HE-AC	236423.3 ± 13377^a^	30430.18 ± 3364^a^	71455.42 ± 8912^a^	164967.9 ± 6554^a^	88.60 ± 5.01^a^	11.40 ± 1.26^b^
HO-SP	216556.1 ± 11493^c^	26504.53 ± 2938^d^	62877.78 ± 6661^d^	153678.4 ± 6011^b^	89.09 ± 4.72^a^	10.91 ± 0.98^d^
HE-SP	228173.8 ± 2826^a^	31864.06 ± 913^a^	71600.37 ± 3170^a^	156573.5 ± 457^a^	87.75 ± 0.35^d^	12.25 ± 1.08^a^

The results are presented mean ± SD (*n* = 3). Different letters indicated that the difference between the groups was statistically significant (*p* < 0.05).

At the end of oxidation, the contents of unsaturated fatty acids, saturated fatty acids, monounsaturated fatty acids, and polyunsaturated fatty acids in walnut oil added with SP increased by 5.09, 16.81, 12.18, and 1.84%, respectively. After adding 35%-SO to walnut oil, the content of unsaturated fatty acid, saturated fatty acid, monounsaturated fatty acid, and polyunsaturated fatty acid in walnut oil increased by 7.29, 4.13, 3.01, and 4.74%, respectively. The content of saturated fatty acid, unsaturated fatty acid, monounsaturated fatty acid, and polyunsaturated fatty acid in walnut oil added with AC increased by 10.51, 10.90, 13.66, and 9.7%, respectively. The contents of saturated fatty acids, unsaturated fatty acids, monounsaturated fatty acids, and polyunsaturated fatty acids in walnut oil added with TBHQ decreased by 8.48, 4.58, 7.76, and 3.20%, respectively. The contents of saturated fatty acids, unsaturated fatty acids, monounsaturated fatty acids, and polyunsaturated fatty acids in walnut oil added with BHA decreased by 5.51, −1.18, 0.61, and −1.91%, respectively. The content of saturated fatty acids, unsaturated fatty acids, monounsaturated fatty acids, and polyunsaturated fatty acids in walnut oil added with citric acid increased by 15.08, 9.95, 15.34, and 7.67%, respectively. The contents of saturated fatty acids, unsaturated fatty acids, monounsaturated fatty acids, and polyunsaturated fatty acids in walnut oil decreased by 21.59, 10.31, 19.77, and 6.11%, respectively. It can be seen from the above that after adding several antioxidants, the change of fatty acid content in walnut oil is lower than that of blank walnut oil, indicating that several antioxidants can inhibit the change of fatty acid content in walnut oil and have antioxidant effect on walnut oil. The addition of SP, 35%-SO, and AC can not only inhibit the decrease of monounsaturated fatty acid and polyunsaturated fatty acid content in walnut oil but also increase the content of monounsaturated fatty acid and polyunsaturated fatty acid, indicating that several antioxidants can inhibit the oxidation of walnut oil, which is conducive in improving the oxidation stability of walnut oil. The effect of AC is greater than 35%-SO and SP.

### 3.4 Correlation analysis between endogenous substances

Because the measured substances are endogenous substances in walnut oil, there is a certain correlation. SPSS18.0 was used to analyze the correlation between saturated fatty acids, unsaturated fatty acids, monounsaturated fatty acids, polyunsaturated fatty acids, main fatty acids, carbonyl compounds, conjugated olefins, peroxide value, acid value and total flavonoids, total polyphenols, total sterols, vitamin E, *β*-carotene, and other indicators in walnut oil (the amount of change from the initial stage of oxidation to the end of oxidation). The correlation coefficient was analyzed by Spearman, and the analysis results were as follows.

There is a certain correlation between the endogenous components in walnut oil. Flavonoids in walnut oil showed a significant negative correlation with carbonyl compounds and a moderate positive correlation with conjugated olefins. Polyphenols were strongly negatively correlated with conjugated trienes, strongly positively correlated with unsaturated fatty acids such as C16:1, C18: 1 N9 C, C18: 1 N7, C20:1, and C18:3 N3, significantly positively correlated with monounsaturated fatty acids, and positively correlated with polyunsaturated fatty acids; vitamin E was strongly negatively correlated with acid value; there was a weak correlation between *β*-carotene and fatty acid content. The above results indicate that the trace components polyphenols, flavonoids, sterols, β-carotene, and vitamin E in walnut oil are related to the stability of walnut oil.

## 4 Conclusion

Based on the changes of endogenous characteristics during the oxidation process, this experiment explored the effects of several natural antioxidants on the stability of walnut oil. The results showed that the antioxidant components polyphenols, flavonoids, sterols, β-carotene, and vitamin E in walnut oil were correlated with the changes of fatty acid content, carbonyl compounds, codiene, acid value, and peroxide value in walnut oil. SP, AC, and 35%-SO can inhibit the increase of carbonyl compounds, peroxide value, acid value, and conjugated olefins in walnut oil, and can reduce the loss of endogenous antioxidants such as polyphenols, flavonoids, sterols, vitamin E, and β-carotene. However, the inhibitory effects are inconsistent, which may be due to the different types of antioxidants. It can also inhibit the change of fatty acid content in walnut oil, and can inhibit the decrease of unsaturated fatty acid content. Even after adding several antioxidants such as SP, AC, and 35%-SO, the content of unsaturated fatty acid in walnut oil is increasing, indicating that several antioxidants have a certain protective effect on unsaturated fatty acids in walnut oil, and improve the quality of walnut oil. Among them, AC and SP inhibited the reduction of sterols, polyphenols and vitamin E, and the stability of monounsaturated fatty acids and polyunsaturated fatty acids was better than other treatments (35% sesame oil, BHA, and citric acid), indicating that AC and SP could improve the stability of walnut oil, and the effect of AC was greater than that of SP. In summary, the addition of several natural antioxidants can enhance the endogenous antioxidant substances of walnut oil, inhibit the oxidation of unsaturated fatty acids, and inhibit the increase of carbonyl compounds, codiene, acid value, and peroxide value, indicating that several antioxidants have antioxidant effects on walnut oil and improve the stability of walnut oil, which concludes that the effect of compound antioxidants is greater than that of other antioxidants.

The results of this study provide a reference for the application of endogenous antioxidant components to improve the oxidative stability of walnut oil, and also provide some scientific guidance for the compound research of natural antioxidants. Sesamol, as an endogenous antioxidant in sesame oil, has a good antioxidant effect on the stability of sesame oil. Sesamol can be selected as a natural antioxidant or a compound antioxidant for oil, which is widely used in the oil antioxidant industry and can reduce the use of artificial antioxidants. However, due to the complexity of vegetable oil components, the concentration and proportion of endogenous antioxidant components may have an impact on the type and degree of interaction, so the multiple interactions between them need to be further studied.

## Data Availability

The original contributions presented in the study are included in the article/supplementary material, further inquiries can be directed to the corresponding author.
